# Duodenal Metabolic Profile Changes in Heat-Stressed Broilers

**DOI:** 10.3390/ani12111337

**Published:** 2022-05-24

**Authors:** Jalila S. Dridi, Elizabeth S. Greene, Craig W. Maynard, Giorgio Brugaletta, Alison Ramser, Courtney J. Christopher, Shawn R. Campagna, Hector F. Castro, Sami Dridi

**Affiliations:** 1 École Universitaire de Kinésithérapie, Université d’Orléans, Rue de Chartres, 45100 Orléans, France; jaliladr2@gmail.com; 2 Center of Excellence for Poultry Science, University of Arkansas, Fayetteville, AR 72701, USA; esgreene@uark.edu (E.S.G.); cwmaynar@uark.edu (C.W.M.); giorgiob@uark.edu (G.B.); atramser@uark.edu (A.R.); 3 Department of Agricultural and Food Sciences, Alma Mater Studiorum, University of Bologna, 40064 Bologna, Italy; 4 Department of Chemistry, University of Tennessee, Knoxville, TN 37996, USA; cleathe3@vols.utk.edu (C.J.C.); campagna@utk.edu (S.R.C.); 5 Biological and Small Molecule Mass Spectrometry Core, University of Tennessee, Knoxville, TN 37996, USA; hcastrog@utk.edu

**Keywords:** heat stress, broilers, metabolomics, canonical pathways, mass spectrometry

## Abstract

**Simple Summary:**

Heat stress (HS) represents an environmental and socio-economic burden to the poultry industry worldwide. However, the underpinning mechanisms for HS responses are still not well defined. Here, we used a high-throughput analysis to determine the metabolite profiles in acute and chronic heat-stressed broilers in comparison with thermoneutral and pair-fed birds. The results showed that HS altered several duodenal metabolites in a duration-dependent manner and identified potential metabolite signatures.

**Abstract:**

Heat stress (HS) is devastating to poultry production sustainability worldwide. In addition to its adverse effects on growth, welfare, meat quality, and mortality, HS alters the gut integrity, leading to dysbiosis and leaky gut syndrome; however, the underlying mechanisms are not fully defined. Here, we used a high-throughput mass spectrometric metabolomics approach to probe the metabolite profile in the duodenum of modern broilers exposed to acute (AHS, 2 h) or chronic cyclic (CHS, 8 h/day for 2 weeks) HS in comparison with thermoneutral (TN) and pair-fed birds. Ultra high performance liquid chromatography coupled with high resolution mass spectrometry (UHPLC–HRMS) identified a total of 178 known metabolites. The trajectory analysis of the principal component analysis (PCA) score plots (both 2D and 3D maps) showed clear separation between TN and each treated group, indicating a unique duodenal metabolite profile in HS birds. Within the HS groups, partial least squares discriminant analysis (PLS-DA) displayed different clusters when comparing metabolite profiles from AHS and CHS birds, suggesting that the metabolite signatures were also dependent on HS duration. To gain biologically related molecule networks, the above identified duodenal metabolites were mapped into the Ingenuity Pathway Analysis (IPA) knowledge-base and analyzed to outline the most enriched biological functions. Several common and specific top canonical pathways were generated. Specifically, the adenosine nucleotide degradation and dopamine degradation pathways were specific for the AHS group; however, the UDP-D-xylose and UDP-D-glucuronate biosynthesis pathways were generated only for the CHS group. The top diseases enriched by the IPA core analysis for the DA metabolites, including cancer, organismal (GI) injury, hematological, cardiovascular, developmental, hereditary, and neurological disorders, were group-specific. The top altered molecular and cellular functions were amino acid metabolism, molecular transport, small molecule biochemistry, protein synthesis, cell death and survival, and DNA damage and repair. The IPA-causal network predicted that the upstream regulators (carnitine palmitoyltransferase 1B, CPT1B; histone deacetylase 11, HDAC11; carbonic anhydrase 9, CA9; interleukin 37, IL37; glycine N-methyl transferase, GNMT; GATA4) and the downstream mediators (mitogen-activated protein kinases, MAPKs; superoxide dismutase, SOD) were altered in the HS groups. Taken together, these data showed that, independently of feed intake depression, HS induced significant changes in the duodenal metabolite profile in a duration-dependent manner and identified a potential duodenal signature for HS.

## 1. Introduction

Poultry meat is highly regarded as an efficient source for high quality proteins with affordable prices and without religious taboos. With a global annual average production of 99,901 × 10^3^ metric tons [1], strengthened by intensive genetic selection and long-term genetic gain (growth rate, breast yield, feed efficiency) and improvement of housing and management, broiler (meat-type) chickens are a central component of the worldwide meat production market and support the livelihoods and food security of billions of people. However, these unprecedented and successful advances were associated with several unexpected and undesirable changes, such as the emergence of metabolic disorders (muscle myopathies, etc.) and hypersensitivity to high environmental temperatures [2,3].

Climate change and global warming are real concerns, as unusually warm hot seasons and temperature anomalies have markedly increased and broadened over the past decades [4,5,6,7,8]. Global warming and heat stress are adversely affecting every biological system, including birds, large animals, insects, and crops, and thereby threatening the sustainability of global agricultural production [8,9,10,11]. Of particular interest, broiler chickens are very susceptible to heat stress, as they lack sweat glands, are covered with feathers, and have high metabolic activity and core body temperatures [12,13,14]. The strong adverse effects of heat stress on poultry performance, well-being, mortality, meat yield and quality are well documented [15,16,17,18,19,20,21]. One of the prominent negative effects of heat stress is gut injury; however, its underlying mechanisms are not well understood [22,23]. The brain–gut axis, involving the enteric nervous system (ENS), the autonomic nervous system (ANS), the hypothalamus–pituitary axis (HPA), and the central nervous system (CNS), is very responsive to any stress, including heat load [24,25,26,27]. Although the complexity of the brain–gut interactions, functionality, and network is still at the beginning of unraveling, there is evidence that heat stress triggers the activation of HPA and ANS, leading to increased corticosterone levels and pro-inflammatory cytokines, which in turn affect the intestinal homeostatic functions [28]. For instance, it has been shown that the abovementioned changes, in combination with depressed feed intake induced by heat stress, alter gut motility, flux patterns, secretory activity, content viscosity, and pH [23,29,30].

It is important to highlight that, to dissipate heat during high ambient temperature, heat-stressed birds divert blood flow to the periphery (skin) [31,32,33,34]. This, in turn, leads to a hypoxia-like state in several internal organs, including the gut [35,36]. Combined with low nutrient supply, which is exacerbated by depression in energy intake, hypoxia would result in adenosine triphosphate (ATP) depletion, promoting oxidative and nitrosative stress that leads to leaky gut syndrome [37,38,39]. The integrity of the intestinal barrier and its effective functionality are vital for the overall health and performance of broilers [40,41,42]. As heat stress is a global socio-economic burden that jeopardizes poultry welfare, profitability, and global food security, and as larger and widespread heat waves are predicted for the next century [43,44,45], there is a critical need to define the mechanisms of heat stress responses and their effect on poultry intestinal permeability.

In previous studies, we have shown that heat stress alters the expression of intestinal heat-shock proteins, cyto(chemo)kines, tight junction proteins, and nutrient transporters, thereby inducing leaky gut syndrome [15,46,47]. To gain further in-depth insights into heat stress responses and intestinal barrier integrity, we undertook the present study using an ultra-high performance liquid chromatography–high resolution mass spectrometry (UHPLC–HRMS) based metabolomics approach to determine the metabolic profile in the duodenum of modern broilers exposed to acute (2 h) or chronic cyclic (8 h/day for 2 weeks) heat stress in comparison with thermoneutral and pair-fed birds.

## 2. Materials and Methods

### 2.1. Ethics Statements

The present study was conducted in accordance with the recommendations in the guide for the care and use of laboratory animals of the National Institutes of Health, and the protocol was approved by the Institutional Animal Care and Use Committee (#21050) at the University of Arkansas.

### 2.2. Birds, Diets, and Heat Stress Challenge

**Figure 1:**
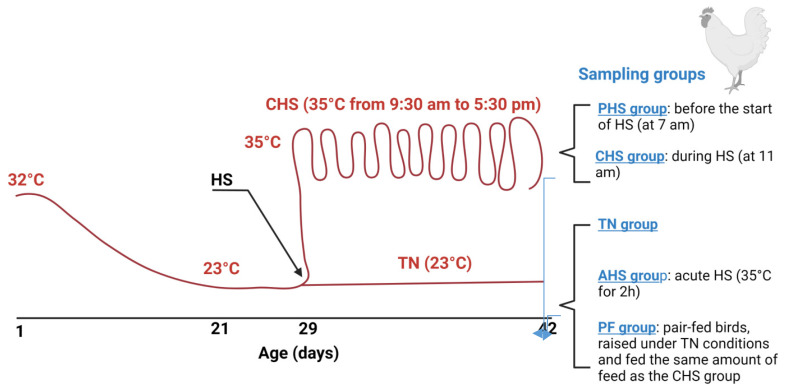
Experimental design representation and heat stress challenge. Temperature was gradually reduced from 32 °C to approximately 23 °C on d 21. At d 29, the experiment followed a completely randomized design with three treatments (8 replicate pens/treatment, 28 birds/pen): a control group (TN) where the birds were raised under thermoneutral condition (23 °C) from d 29–42, a chronic cyclic heat-stressed group (CHS) where the birds were exposed to high ambient temperature (35 °C) for 8 h/d (9:30 am to 5:30 pm) from d 29–42, and a pair-fed group (PF) where the birds were raised like the control group (similar environmental conditions, 23 °C) and fed the same amount of feed as the CHS group. Two additional groups were also used: an acute heat-stressed group (AHS) where some TN birds were exposed to 35 °C for 2 h before sampling on d 42, and a preheat-stressed group (PHS) where CHS birds were sampled before starting the heat stress on d 42. AHS, acute heat stress; CHS, chronic cyclic heat stress; HS, heat stress; PHS, preheat stress; PF, pair fed.

One-day old male broiler (meat-type) chicks (*n* = 672) were obtained from commercial Cobb-Vantress hatchery (Siloam Springs, Arkansas), neck tagged, individually weighed, and randomly allocated to 12 environmental chambers (2 floor pens/chamber, 24 pens in total, 28 birds/pen). Each pen was covered with 7 cm fresh pine shavings and equipped with a plastic hanging poultry feeder and an automatic vacuum-sealing O-ring drinker. Birds were given *ad libitum* access to clean and fresh water and a corn–soybean meal basal diet (starter d 1–14, grower d 15–28, and finisher d 29–42). The diet composition has been previously described [15]. Temperature was maintained at 32 °C for the first 3 days, and then gradually reduced approximately 3 °C each week until it reached 23 °C on d 21. The average relative humidity was 30%. The lighting program was 24 h light for the first 3 days, reduced to 23 h light:1 h dark during d 4–7, and reduced further to 18 h light: 6 h dark thereafter. The experiment followed a completely randomized design with three treatments (8 replicate pens/treatment): a control group (TN) where the birds were raised under thermoneutral condition (23 °C) from d 29–42, a chronic cyclic heat-stressed group (CHS) where the birds were exposed to high ambient temperature (35 °C) for 8 h/d (9:30 am to 5:30 pm) from d 29–42 to mimic summer time in Arkansas, and a pair-fed group (PF) where the birds were raised like the control group (similar environmental conditions, 23 °C) and fed the same amount of feed as the CHS group. Using the pair-fed TN group would help to distinguish between the effect of feed depression and the effect of heat stress. Feed intake and water consumption were recorded daily. Individual body weight was recorded weekly. Body core temperature was continuously monitored using Thermochron temperature logger (iButton, DS19221, Embedded Data Systems, Lawrenceburg, KY, USA). The environmental temperature and humidity were also continuously recorded in each chamber. At the end of the experiment (d 42), duodenum segments from each group (TN, CHS, PF, *n* = 8/group) were collected, rinsed in PBS 1x, snap frozen in liquid nitrogen, and stored at −80 °C for metabolomics analysis. Duodenum sections were also collected from two additional groups: an acute heat-stressed group (AHS), where some TN birds were exposed to 35 °C for 2 h before sampling on d 42, and a preheat-stressed group (PHS), where CHS birds were sampled before starting the heat stress on d 42 (Figure 1).

### 2.3. Sample Collection and Preparation

Duodenum tissues were ground, snap frozen in liquid nitrogen, and sent to the Biological and Small Molecule Mass Spectrometry Core (BSMMSC, The University of Tennessee, Knoxville, TN, USA). Metabolites were extracted with 1.5 mL of extraction solvent (40:40:20 HPLC grade methanol: acetonitrile: water with formic acid at a final concentration of 0.1 M), pre-chilled at 4 °C, and incubated at −20 °C for 20 min. Samples were centrifuged (13,300× *g*, 5 min, 4 °C), and supernatants were collected. Solvent was evaporated under a stream of nitrogen, and metabolites were suspended with 300 µL of HPLC-grade water prior to mass analysis.

### 2.4. Ultra-High Performance Liquid Chromatography—High Resolution Mass Spectrometry (UHPLC–HRMS) Metabolomics Analysis

UHPLC–HRMS analysis has been described previously [48,49]. Briefly, metabolites were separated on a Dionex UltiMate 3000 RS (Sunnyvale, CA, USA) by injecting a 10 µL sample on a Synergy reverse phase Hydro-RP 100 Å, 100 mm × 2.00 mm, 2.5 µm pore size LC column (Phenomenex, Torrance, CA, USA) kept at 25 °C. The global metabolomics method, adapted from [50], ran for 26 min with the application of a multistep gradient. To separate the analytes, two HPLC-grade solvents were used in gradient steps. Solvent A (97:3 H_2_O:MeOH with 11 mM tributylamine and 15 mM acetic acid) and solvent B (100% MeOH). The gradient was performed as follows: 0 min, 0% B; 5 min, 20% B; 13 min, 55% B; 15.5 min, 95% B; 19 min, 0% B; 25 min, 0% B with a flow rate of 200 μL/min. The eluent was administered into the mass spectrometer via an electrospray ionization (ESI) source conjoined to an Exactive™ Plus Orbitrap Mass Spectrometer (Thermo Scientific, Waltham, MA, USA) under the following established parameters of aux gas: 8; sheath gas: 25; sweep gas: 3; spray voltage: 3.00 kV; and capillary temperature: 300 °C. The parameters of the mass spectrometer were set as follows: resolution: 140,000; automatic gain control (AGC): 3 × 106; maximum IT time: 100; scan range: 85–1000 *m*/*z*. Raw data were obtained from the Xcalibur MS software (Thermo Electron Corp, Waltham, MA, USA) and converted to mzML format by ProteoWizard tool MSConverter [51,52]. The converted data were analyzed using MAVEN [53], and peaks were annotated with a maximum allowed error of 5 ppm. Area under the chromatographic curve was integrated based upon an in-house verified list of metabolites using exact mass and known retention times [54]. All metabolite values were normalized based on the mass of the duodenum tissue extracted prior to all statistical calculations.

### 2.5. Ingenuity Pathway Analysis (IPA)

The metabolites’ information (fold change and *p*-value) as well as their IDs (Human Metabolome Database, HMDB [55]; Kyoto Encyclopedia of Genes and Genomes, KEGG [56]; and Chemical Entities of Biological Interest, ChEBI [57]) were introduced for the IPA analysis to determine the canonical pathways, functional annotation, upstream and downstream analysis, and molecular network discovery using a cut-off of FDR adjusted *p*-value < 0.05 and a fold change between −1.5 and 1.5.

### 2.6. RNA Isolation and Quantitative Real-Time PCR

Isolation, integrity assessment, and concentration measurement of duodenal total RNA were previously described [58,59,60,61]. Duodenal total RNA samples were DNase treated, reverse-transcribed using qScript cDNA Synthesis Supermix (Quanta Biosciences, Gaithersburg, MD, USA), and amplified by real-time quantitative PCR (Applied Biosystems 7500 Real Time System) with PowerUp SYBR green master mix (Life Technologies, Carlsbad, CA, USA) as previously described [58,59,60,61]. The qPCR cycling conditions and melt curve analysis were previously reported [58,59,60,61]. Relative expression of the target genes was determined using the 2^−ΔΔCT^ method, with normalization to 18s rRNA as a housekeeping gene [62]. Oligonucleotide primer sequences specific for chicken are presented in Table 1.

### 2.7. Immunoblot Analysis

Duodenal tissue homogenization, protein isolation, and protein concentration measurement using Bradford assay and Synergy HT multimode microplate reader (BioTek, Winooski, VT, USA) were previously described [63]. Proteins (80 μg) were run on 4–12% gradient Bis–Tris gels (Life Technologies, Carlsbad, CA, USA) and transferred to PVDF membranes. The membranes were blocked with 5% non-fat milk in tris-buffered saline with Tween 20 detergent (TBS-T) for 1 h at room temperature, then incubated with primary antibodies (1:500–1:1000 dilution) overnight at 4 °C. Secondary antibodies (1:5000) were diluted in 5% milk in TBS-T, and membranes were incubated at room temperature for 1 h. Primary antibodies used were: rabbit anti-phospho-ERK1/2^Thr202^/^Tyr204^, rabbit anti-ERK1/2, rabbit anti-phospho-P38 MAPK^Thr180^/^Tyr182^, rabbit anti-P38 MAPK (Cell Signaling Technology, Danvers, MA, USA), and rabbit anti-GAPDH antibody (Santa Cruz Biotechnology, Dallas, TX, USA) as a housekeeping protein to assess protein loading. After another wash, secondary anti-rabbit IgG and HRP-linked antibody (Cell Signaling Technology, Danvers, MA) diluted to 1:5000 were added to 5% nonfat milk in TBS and Tween 20 and incubated with the membranes at room temperature for 1 h. The signal was visualized by chemiluminescence (ECL Plus, GE Healthcare, Pittsburg, PA, USA) and captured by the FluorChem M MultiFluor System (ProteinSimple, San Jose, CA, USA).

### 2.8. Data Processing and Statistical Analysis

Original datasets from all studied groups have been submitted to EMBL-EBI MetaboLights database, DOI: 10.1093/nar/gkz1019, PMID:31691833) with the identifier MTBLS4513 (https://www.ebi.ac.uk/metabolights/MTBLS4513) (access on 20 March 2022). Metabolites showing differences higher or lower than 1.5 folds and *p*-value less than 0.05 in the comparison between each of the treatment groups (AHS, PHS, CHS, and PF) and TN (control) birds were considered differentially abundant. Heat maps, which displayed log_2_ fold changes for identified metabolites, were created with Cluster 3.0 [64] and Javatreeview 1.1 [65]. *p*-values were calculated using Student’s *t*-test or Student–Newman–Keuls (SNK) method as appropriate. For group discrimination, partial least squares discriminant analysis (PLS-DA) and variable importance in projection (VIP) scores were constructed using the MetaboAnalyst 5 [66] and statistical package DiscriMiner in R version 3.6.1 (https://cran.r-project.org) (access on 20 March 2022). Metabolites with VIP values > 1 were the ones that contributed to the group differentiation, and this was considered as a significant VIP score.

## 3. Results

### 3.1. Global Analysis of the Duodenal Dynamic Metabolic Profiling

**Figure 2:**
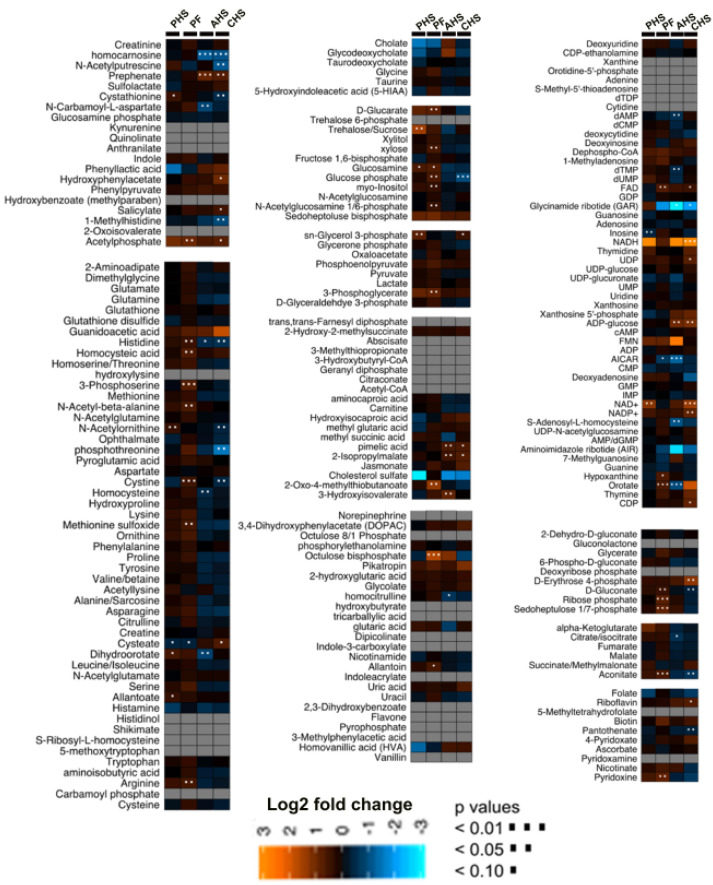
Heat map of relative levels of broiler duodenal metabolites modulated by heat stress exposure. Heat map was built using log2 fold change and VIP score and by comparing each treatment (PHS, AHS, CHS, and PF) to the TN group. AHS, acute heat stress; CHS, chronic heat stress; PF, pair fed; PHS, pre-heat stress group. The empty cells indicate that the metabolites were not detected.

The global metabolomics profiling analyses identified a total of 218 metabolites with 178 that were highly abundant, while 40 were not detectable in broiler duodenum. The heat map provides a general overview of the identified metabolite relative abundances for each treatment group compared to TN group (Figure 2).

**Figure 3:**
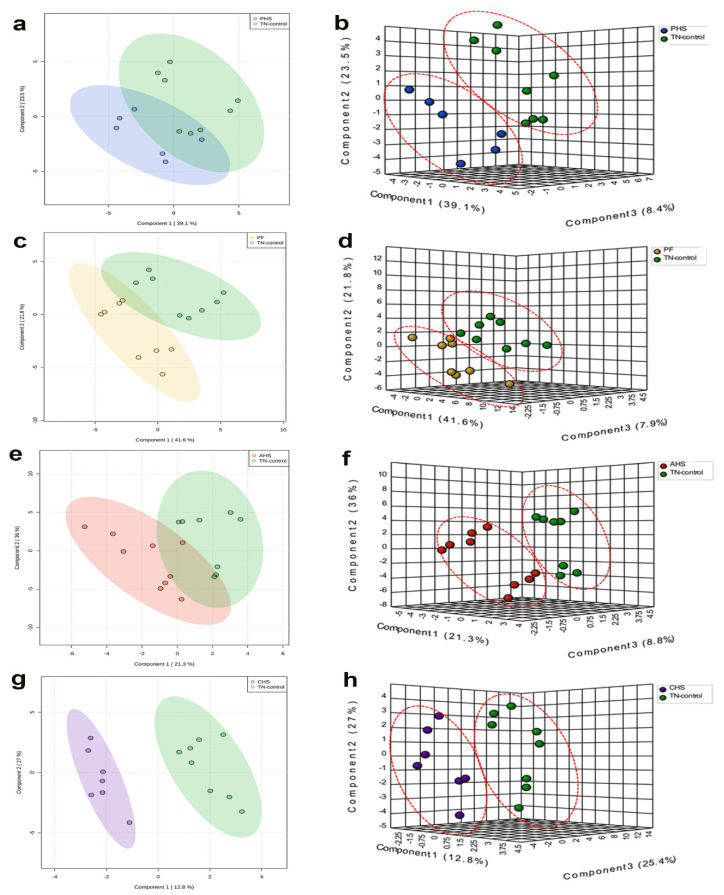
Partial least squares-discriminant analysis (PLS-DA) 2D and 3D score plots. PLS-DA was constructed using the statistical package DiscriMiner in R version 3.6.1 and displayed different clusters when comparing metabolite profiles between TN and treated (CHS, AHS, PF, and PHS) birds (**a**,**c**,**e**,**g**). AHS (**f**), acute heat stress; CHS (**h**), chronic heat stress; PF (**d**), pair fed; PHS (**b**), preheat stress; TN, thermoneutral.

The 178 identified metabolites have been submitted to MetabolLights database (https://www.ebi.ac.uk/metabolights) (access on 20 March 2022) and are presented in Table S1. As depicted in Figure 3, the only treatment with clear separation from the TN group in the 2D PLS-DA plot was the CHS group (Figure 3g). However, the 3D PLS-DA plots showed clear separation between TN and each of the PHS (Figure 3b), PF (Figure 3d), AHS (Figure 3f), and CHS (Figure 3h) groups.

**Figure 4:**
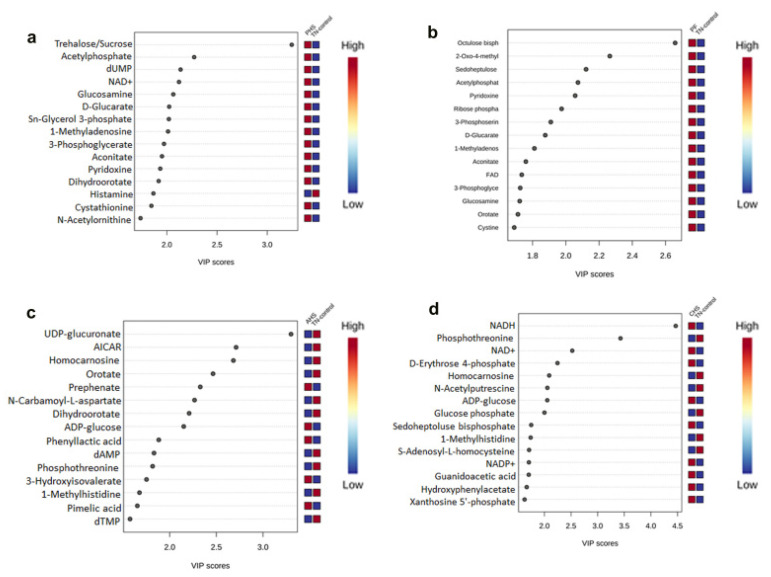
VIP scores and related concentrations of the differentially abundant metabolites. Top 15 metabolites based on VIP scores from PLS-DA analysis comparing TN to PHS (**a**), TN to PF (**b**), TN to AHS (**c**), and TN to CHS (**d**). AHS, acute heat stress; AICAR, 5-aminoimidazole-4-carboxamide-1-β-D-ribofuranoside; CHS, chronic heat stress; dAMP, deoxyadenosine monophosphate; dTMP, deoxythymidine monophosphate; dUMP, deoxyuridine monophosphate; NAD^+^, nicotinamide adenine dinucleotide; NADH, reduced nicotinamide adenine dinucleotide; PHS, preheat stress; PF, pair fed; TN, thermoneutral.

When all of the groups were plotted together, the segregation was less clear (data not shown). In order to determine which metabolites drove the separation between the groups, each metabolite was assigned a VIP score representing its importance to the PLS-DA model with a VIP score >1 reflecting a significant influence on the separated clusters observed. As shown by the VIP score plot, the 15 metabolites with the highest VIP scores were identified for each comparison (Figure 4a–d).

### 3.2. Identification of Potential Metabolic Signatures

For more stringent analyses, metabolites with the greatest discriminating potential between the thermoneutral (TN) and each of the treated-groups (PHS, PF, AHS, or CHS) were assessed by unpaired *t*-test with significance level *p* < 0.05. As shown in Table 2, metabolites were clustered in a group-specific manner. For instance, cystathionine, N-acetylornithine, dihydroorotate, allantoate, and trehalose were specifically and significantly abundant in the duodenum of PHS group (Table 2). Acetylphosphate, histidine, homocysteic acid, 3-Phosphoserine, N-Acetyl-beta-alanine, cystine, methionine sulfoxide, arginine, D-glucarate, xylose, glucose phosphate, myo-inositol, N-acetylglucosamine 1/6-phosphate, 3-Phosphoglycerate, 2-oxo-4-methylthiobutanoate, hypoxanthine, orotate, octulose bisphosphate, allantoin, D-gluconate, ribose phosphate, sedoheptulose 1/7-phosphate, aconitate, and pyridoxine were significantly high in the duodenum of the PF group (Table 2). 3-Hydroxyisovalerate was specifically high in the AHS group only; however, hydroxyphenylacetate, salicylate, NADH, UDP, NADP+, CDP, D-erythrose 4-phosphate, and riboflavin were abundant in the duodenum of the CHS group (Table 2). On the other hand, inosine was significantly decreased in PHS duodenum; however, N-carbamoyl-L-aspartate, homocysteine, dAMP, dTMP, S-adenosyl-L-homocysteine, homocitrulline, and citrate were significantly low in the duodenum of the AHS group (Table 2). N-acetylputrescine, 1-methylhistidine, phosphothreonine, and pantothenate were specifically depressed in the duodenum of the CHS group (Table 2).

It is worth noting that the levels of some duodenal metabolites followed opposite patterns between groups. For example, cystathionine was abundant in the PHS group but depressed in the CHS group (Table 2). Dihydroorotate was high in the PHS but low in the AHS group. Cysteate was decreased in PHS and PF but increased in the CHS group (Table 2). Histidine was high in the PF but low in the AHS and CHS groups. Cystine, glucose phosphate, and D-gluconate were increased in the PF but decreased in the CHS group. Orotate was increased in the PF but decreased in the AHS group (Table 2). The differential abundance of these metabolites indicated a specific and distinct metabolic pattern between TN and treated groups, and within treated groups.

### 3.3. Metabolic Pathway and Network Analysis

#### 3.3.1. Top Canonical Pathways

To gain biologically related molecule networks, the above identified metabolites (172 from 178) were mapped into the IPA knowledge-base and analyzed to outline the most enriched biological functions. Using a cut-off of FDR adjusted *p*-value <0.05 and a fold-change between −0.5 and 0.5, several common and specific top canonical pathways were generated (Table 3). The adenosine nucleotide degradation and dopamine degradation pathways were specific to the AHS group (Table 3). The UDP-D-xylose and UDP-D-glucuronate biosynthesis pathways were generated only for the CHS group (Table 3); however, the tRNA charging, pyruvate fermentation to lactate, and Glycerol-3-phosphate shuttle pathways were specific to the PHS birds. The sirtuin signaling pathway and gluconeogenesis pathway were specific to the PF group (Table 3). The pathways associated with purine nucleotide degradation, urate biosynthesis/inosine 5′-phosphate degradation, and ascorbate recycling were common for both the AHS and CHS (Table 3). The salvage pathways of pyrimidine deoxyribonucleotides were identified in both CHS and PF groups (Table 3). The glycine betaine degradation and creatine biosynthesis pathways were generated for both the PHS and PF groups (Table 3). Within the common purine nucleotide degradation pathway, hypoxanthine was specific to AHS, but adenosine and GMP were specific to the CHS group (Table 3). In the ascorbate recycling pathway, glutathione was specific to the CHS but not the AHS group (Table 3). In the salvage pathways of pyrimidine deoxyribonucleotides, deoxycytidine and uracil were specific to the PF group (Table 3). Similarly, within the creatine biosynthesis pathway, s-adenosylhomocysteine was specific to the PF group (Table 3).

#### 3.3.2. Top Diseases and Disorders

**Figure 5:**
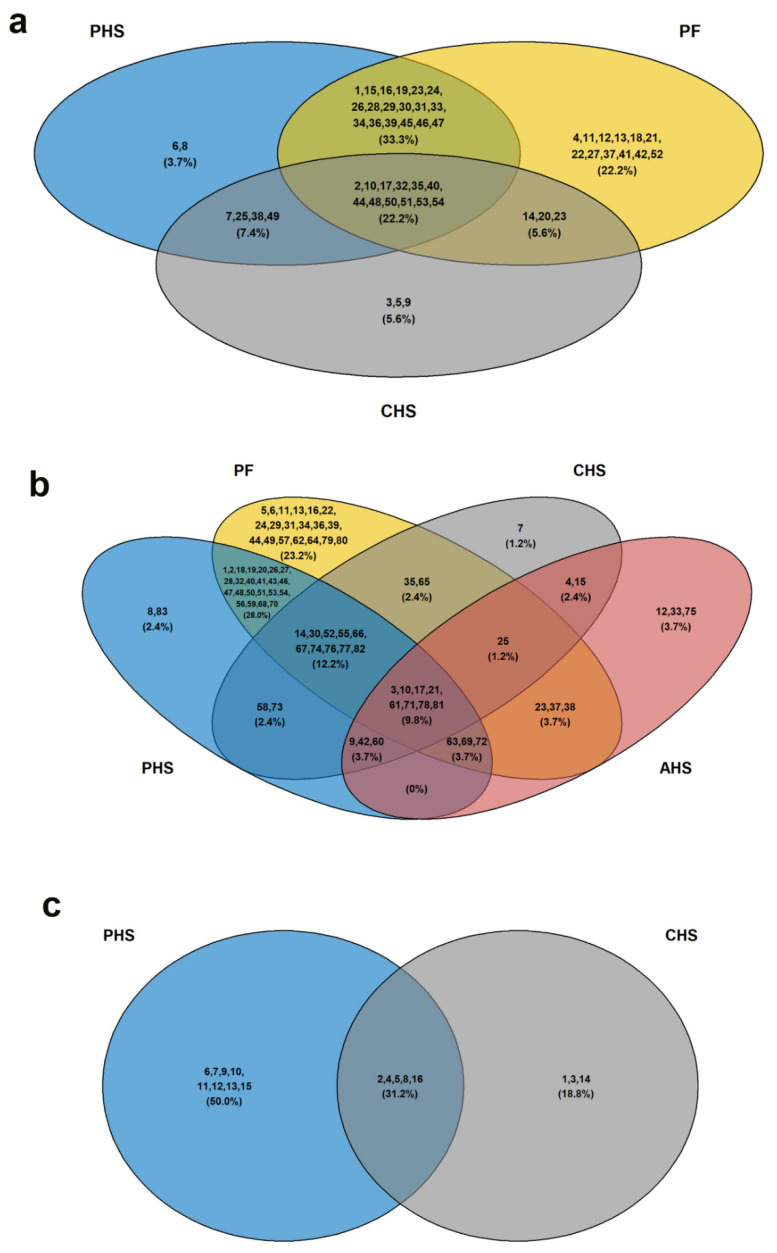
Venn diagrams showing the overlap of duodenal metabolites in top diseases enriched by IPA analysis. (**a**) Cancer, (**b**) organismal (GI) injury, and (**c**) hematological disorder. The list of the molecules is presented in Supplementary Table S1.

The top diseases and disorders enriched by the IPA core analysis for the differentially abundant (DA) metabolites were ranked by *p*-value and summarized in Table 4. Cancer was a common disorder enriched with 36, 22, and 45 metabolites (molecules) in the PHS, CHS, and PF groups, respectively (Table 4). The 3-Venn diagrams showed that 33.3, 22.2, 7.4, and 5.6% of the total metabolites were common between PHS-PF, PHS-PF-CHS, PHS-CHS, and CHS-PF, respectively (Figure 5a). The metabolites AICAR and creatine were specific to PHS; 4-hydroxy-3-methoxyphenylacetic acid, adenosine, and cAMP were specific to CHS; and 5-hydroxyindol-3-acetic acid, folic acid, fructose-1,6-diphosphate, fumaric acid, glutathione disulfide, guanosine, inosine, L-cysteine, malic acid, niacinamide, ophthalmic acid, and uracil were specific to the PF group (Figure 5a). The second common disorder enriched was organismal injury, including GI abnormality, with 51, 23, 29, and 70 molecules in the PHS, AHS, CHS, and PF groups, respectively (Table 4). The 4-Venn diagrams revealed that 28, 12.2, 9.8, 3.7, 3.7, 3.7, 2.4, 2.4, 2.4, and 1.2% of the total metabolites were common between PHS-PF, PHS-PF-CHS, PHS-PF-CHS-AHS, PHS-CHS-AHS, PHS-PF-AHS, PF-AHS, PHS-CHS, PF-CHS, CHS-AHS, and PF-CHS-AHS, respectively (Figure 5b). AICAR and xylitol were specific to PHS, adenosine was distinct for CHS; however, cholic acid, glycodeoxycholic acid, and taurodeoxycholic acid were particular for the AHS group (Figure 5b). Hematological disorder was enriched in both PHS and CHS, with 13 and 8 molecules, respectively (Table 4). Venn diagrams showed that adenosine, cAMP, and oxaloacetic acid were specific to CHS; however, L-arginine, L-asparagine, L-carnitine, L-homocysteine, L-methionine, myo-inositol, nicotinic acid, and pyridoxine were specific to the PHS group (Figure 5c).

Hepatic system disease was enriched only in PHS with 22 molecules; ophthalmic, cardiovascular, developmental, and hereditary disorders were enriched only in AHS, with 3, 3, 4, and 6 molecules, respectively (Table 4). Neurological and psychological disorders were enriched only in CHS; however, inflammatory disease and responses were enriched in PF only (Table 4).

#### 3.3.3. Top Molecular and Cellular Functions

**Figure 6:**
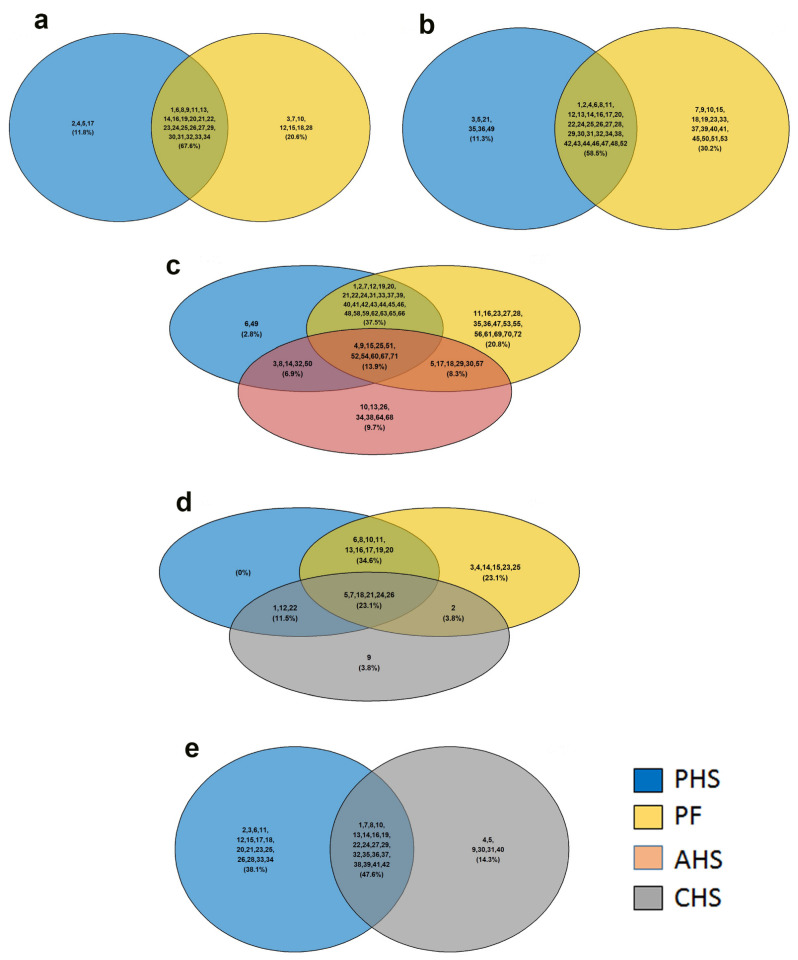
Venn diagrams showing the overlap of duodenal metabolites in top molecular and cellular functions enriched by IPA analysis. (**a**) Amino acid metabolism, (**b**) molecular transport, (**c**) small molecule biochemistry, (**d**) protein synthesis, and (**e**) cell death and survival. The list of the molecules is presented in supplementary Table S1 AHS, acute heat stress; CHS, chronic heat stress; PHS, preheat stress; PF, pair fed.

The ten top molecular and cellular functions were enriched by the IPA core analysis for the DA metabolites, ranked by *p*-value, and summarized in Table 5. Amino acid metabolism and molecular transport were enriched in both PHS and PF groups (Table 5). For the amino acid metabolism, 67.6% of total metabolites were common between the PHS and PF groups; however, 20% (adenine-riboflavin dinucleotide, citrulline, folic acid, glutathione disulfide, guanosine, L-cysteine, oxalacetic acid) were specific to PF, and 11.8% (2-oxoglutaric acid, AICAR, ascorbic acid, L-aspartic acid) were particular to the PHS group (Figure 6a). For molecular transport, 58.5% of total metabolites were common between the PHS and PF groups, while 11.3% (AICAR, ascorbic acid, L-aspartic acid, N-acetylputrescine, NAD^+^, UDP) and 30.2% (citrulline, D-pantothenic acid, folic acid, glutathione disulfide, guanosine, hypoxanthine, L-cysteine, malic acid, niacinamide, orotic acid, oxalacetic acid, phosphoenolpyruvate, S-adenosylhomocysteine, UDP-D-glucose, UMP, uridine) were specific to PHS and PF, respectively (Figure 6b).

Small molecule biochemistry was enriched in PHS, AHS, and PF with 44, 28, and 58 molecules, respectively (Table 5). As shown by Venn diagram, 37.5%, 13.9%, 8.3%, and 6.9% were common between PHS-PF, PHS-PF-AHS, AHS-PF, and PHS-AHS, respectively (Figure 6c). The group-specific enriched metabolites were AICAR and N-acetylputrescine for PHS, cholic acid, cAMP, glycodeoxycholic acid, L-cysteic acid, L-homocysteic acid, taurodeoxycholic acid, UDP-D-glucose for AHS, and citrulline, folic acid, glutathione disulfide, guanosine, homocarnosine, L-cysteine, L-cystine, malic acid, niacinamide, orotic acid, oxalacetic acid, S-adenosylhomocysteine, UDP-N-acetylglucosamine, UMP, and uridine for the PF group (Figure 6c).

Protein synthesis was enriched in PHS, CHS, and PF groups with 18, 11, and 22 molecules, respectively (Table 5). As shown in Figure 6d, the Venn diagram revealed that L-alpha-hydroxyisocaproic acid was specific to the CHS group; however, cholesterol sulfate, citrulline, L-cysteine, L-cystine, niacinamide, and taurine were specific to the PF group.

Cell death and survival was enriched in both the PHS and CHS groups, with 36 and 26 molecules, respectively (Table 5). Venn diagram showed that 3,4-dihydroxyphenylacetic acid, adenosine, cAMP, orotic acid, oxalacetic acid, and UDP-D-glucose were specific to the CHS group; however, 2-deoxyadenosine, 2-oxoglutaric acid, AICAR, deoxycytidine, glucosamine, glycine, L-arginine, L-asparagine, L-carnitine, L-histidine, L-methionine, L-proline, L-serine, L-tyrosine, pyridoxine, and pyruvic acid were specific to the PHS group (Figure 6e).

Nucleic acid metabolism, DNA replication, damage, and repair, energy production, and carbohydrate metabolism were enriched in the AHS group; however, free radical production and scavenging was refined in the CHS group (Table 5).

#### 3.3.4. Top Up- and Downstream Regulators

**Figure 7:**
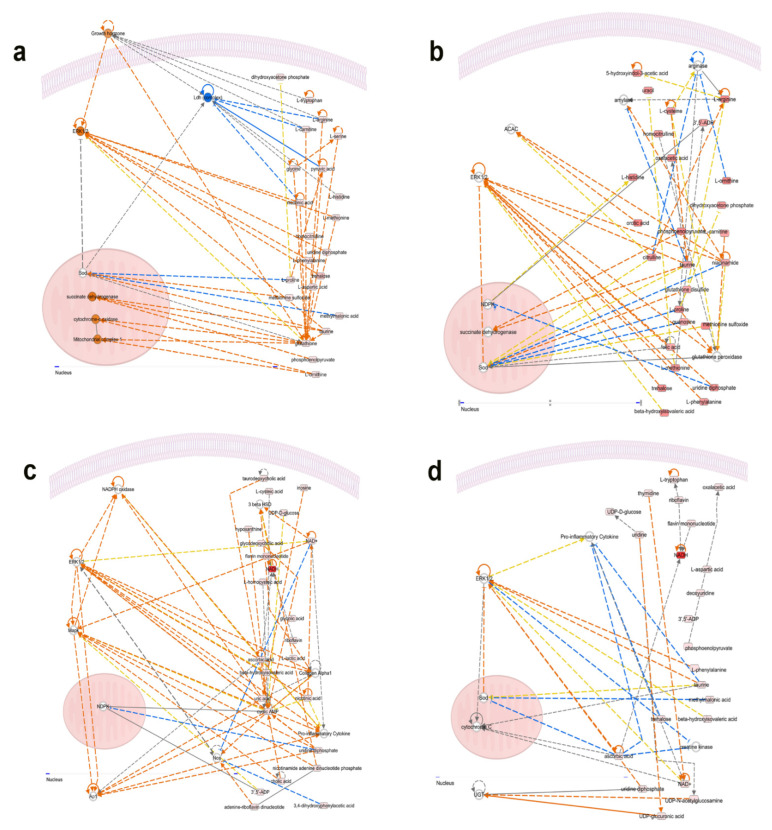
Predicted causal networks and downstream mediators built with IPA program from duodenal metabolomics data. (**a**) PHS group, (**b**) PF group, (**c**) AHS group, and (**d**) CHS group. AHS, acute heat stress; AP1, activator protein 1; CHS, chronic heat stress; ERK, extracellular signal-regulated kinase; MAPK, mitogen-activated protein kinase; NDPK, nucleoside diphosphate kinase; PF, pair fed; PHS, preheat stress; SOD, superoxide dismutase; UGT, UDP-glucuronosyltransferase.

To mine the duodenal metabolome data further, we next assessed how the DA metabolites interacted with intracellular or transcellular signaling and molecules. The integrated IPA-predicted upstream regulators are summarized in Table 6. In the PHS group, glycine N-methyl transferase (GNMT) and carnitine palmitoyltransferase 1B (CPT1B) were inhibited, while histone deacetylase 11 (HDAC11), carbonic anhydrase 9 (CA9), GATA binding protein 4, and matrix metallopeptidase 11 (MMP11) were activated. In CHS birds, CPT1B and IL37 were predicted to be inhibited; however, CA9 was activated. In the AHS group, both CPT1B and cystathionine gamma-lyase (CTH) were predicted to be inhibited. In the PF group, GNMT, IL37, and CPT1B were projected to be inhibited, and CA9, GATA4, and HDAC11 were activated (Table 6). We extended the IPA analyses to predict the downstream mediators, and as shown in Figure 7, the causal network indicated that MAPK and mitochondrial enzymes were central subsequent mediators. The extracellular signal-related kinase 1/2 (ERK1/2) was a central hub for all of the tested groups (Figure 7a–d). The mitochondrial superoxide dismutase (SOD) and cytochrome C oxidase were scored in both the PHS and CHS birds. The mitochondrial succinate dehydrogenase was inferred in the PHS and PF groups. The mitochondrial NDP kinase was predicted in the PF and AHS birds.

**Figure 8:**
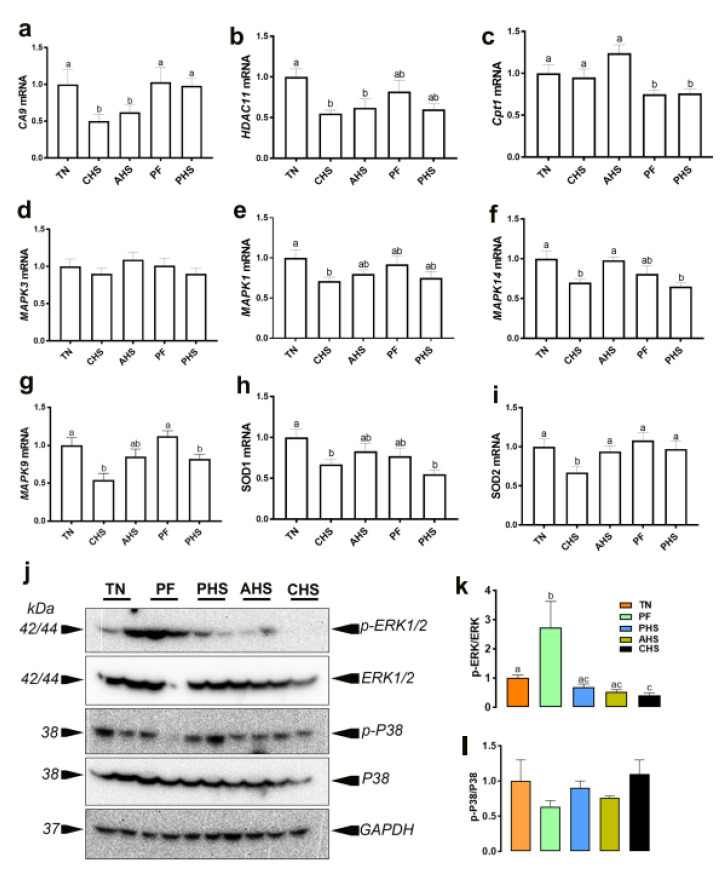
Effect of heat stress on the expression of SOD and MAPKs. The mRNA levels of *CA9* (**a**), *HDAC11* (**b**), *Cpt1* (**c**), *MAPK3* (**d**), *MAPK1* (**e**), *MAPK14* (**f**), *MAPK9* (**g**), *SOD1* (**h**), and *SOD2* (**i**) were measured by real-time qPCR and 2^−ΔΔCt^ method [62]. The protein levels were determined by Western blot (**j**), and the relative expression was presented as phospho protein/ pan protein ratios (**k**,**l**). Data are presented as mean ± SEM (*n* = 8 and 4/group for qPCR and Western blot, respectively). Different letters are significantly different (*p* < 0.05). AHS, acute heat stress; CA9, carbonic anhydrase 9; CHS, chronic heat stress; CPT, Carnitine palmitoyltransferase; GAPDH, glyceraldehyde-3-phosphate dehydrogenase; HDAC 11, histone deacetylase 11; ERK, extracellular signal-regulated protein kinase; MAPK, mitogen-activated protein kinase; PF, pair fed; PHS, preheat stress; SOD, superoxide dismutase.

The mitochondrial complex I was found in the PHS group. At the nuclear level, the activator protein 1 (AP-1) and UDP-glucuronosyltransferase (UGT) were mapped in AHS and CHS, respectively. QPCR analyses of a few selected genes showed that the duodenal expression of *CA9* and *HDAC11* was downregulated in the CHS and AHS group, and *CPT1* in the PF and PHS birds (Figure 8a–c). The expression of duodenal *MAPK14* (P38 MAPK), *MAPK9* (JNK2), and *SOD1* was downregulated in the CHS and PHS groups compared to the TN birds (Figure 8f,h). The expression of *MAPK1* (ERK2) and *SOD2* was downregulated in the CHS compared to TN group (Figure 8e,i); however, the *MAPK3* (ERK1) mRNA abundances remained unchanged between all studied groups (Figure 8d). Western blot analysis, on the other hand, showed that the phosphorylated levels of ERK1/2 at the Thr202/Tyr204 site was significantly induced in PF but decreased in the CHS group compared to TN birds (Figure 8j,k); however, p-P38^Thr180/Tyr182^ did not differ between all groups (Figure 8j,l).

## 4. Discussion

High environmental heat load is one of the most challenging stressors and a stumbling block to the poultry industry as it disrupts production sustainability and inflicts heavy economic burdens worldwide [7,67]. In addition to its strong adverse effects on appetite, growth, performance, welfare, and meat quality [15,16,17,18,68], heat stress can damage body systems and organs [69,70,71]. Although the underlying molecular mechanisms are not fully defined, one of the prominent effects of heat stress is gut injury [28,35,36,72]. As a continuation of our previous researches showing that heat stress alters the expression of intestinal heat-shock proteins, cyto(chemo)kines, tight junction proteins, and nutrient transporters, and thereby induces leaky gut syndrome [15,46,47], we undertook the present study to determine the duodenal metabolic profiles in heat-stressed broilers.

Due to its high resolution and sensitivity, UHPLC–HRMS-based metabolomics has become the leading means of systems biology research and has been extensively used in the biomedical, pharmaceutical, and toxicological fields [73], but its implementation is not prominent in livestock and poultry research. To our knowledge, this is the first study to explore the chemical constituents and to systematically identify DA metabolites in the duodenum of heat-stressed broilers. The results showed that heat stress exposure induced significant changes in the broiler duodenal metabolome independently of feed depression, as the PF group exhibited very minimal overlap with the AHS group and a complete separation from the CHS group. This validates that exposure to heat stress rather than differences in feed intake lead to the different metabolic profiles. Specifically, prephenate, pimelic acid, 2-isopropylmalate, and ADP-glucose were found to be significantly higher in the duodenum of both AHS and CHS birds compared to TN birds. Prephenate is involved in aromatic amino acid biosynthesis and the shikimate pathway, which is present in bacteria, fungi, plants, some parasitic protozoans, and algae, but not in animals [74,75]. Similarly, pimelic acid, also known as heptanedioic acid, is synthesized in many bacteria and is involved in the biosynthesis of lysine and biotin [76]. 2-Isopropylmalate is involved in the leucine biosynthesis pathway, which is common to prokaryotes, plants, and fungi, but absent from human and animals [77,78]. ADP-glucose, however, exists in all living organisms, ranging from bacteria to humans, and is involved in the ticlopidine metabolic pathway. These data suggest that prephenate, pimelic acid, and 2-isopropylmalate are probably microbiota-generated metabolites; however, ADP-glucose could be either microbiota- or host-derived. Although the exact identity of the bacterial/microorganism sources and species is not known at this time, it seems that heat stress modifies the intestinal microbiota- and host-dependent derivative metabolites that potentially play roles as important physiological modulators in stress responses, whether associated with dysbiosis and leaky gut or thermo-memory and adaptation [79,80].

It is worth noting that within heat stress groups and independently of feed intake reduction, there was separation based on heat stress duration, with AHS overlapping with PF, PHS, TN, and CHS, while CHS only showed overlap with AHS in the 3D PLS-DA. This demonstrates that the increased duration of HS further modulates the duodenal metabolome. In fact, 3-hydroxyisovalerate was higher only in the AHS group; however, hydroxyphenylacetate, salicylate, NADH, UDP, NADP^+^, CDP, D-erythrose 4-phosphate, and riboflavin were elevated in CHS birds. 3-Hydroxyisovalerate is a byproduct of the leucine degradation pathway and is a host-derived mitochondrial metabolite through the action of the biotin-dependent enzyme methylcrotonyl-CoA carboxylase [81]. Its high level was associated with mitochondria toxicity, redox dyshomeostasis, aciduria, and ketogenesis disorders [82,83], most of which are induced by acute heat stress [84,85]. The CHS-associated metabolites were both microbiota- and host-derived. Hydroxyphenylacetate has been shown to have a protective ability via induction of antioxidant enzyme activities in mice [86]. Salicylate was found to be central to the defense mechanism in plants [87]. In animals, salicylate inhibits cyclooxygenase-2 transcription, which has been shown to be associated with stress [88,89,90], including heat load [91]. In addition to mitochondrial origin, the observed high levels of NADP^+^, which were in agreement with previous studies [92], suggest that CHS induced reactive oxygen species (ROS) via the nicotinamide adenine dinucleotide phosphate oxidase pathway [92] that converts NADPH to NADP^+^ [93]. NADP^+^ can also be generated from phosphorylated NAD^+^ by the action of NAD^+^ kinases (NNT and NADKs). NADH is a central hydride donor that drives the mitochondrial oxidative phosphorylation (OXPHOS) for ATP generation along with ROS production [94] and the conversion of lactic acid to pyruvate. Although it is not known what subcellular compartment (cytosol, mitochondria, or both) is involved, the high level of NADH supports the abovementioned hypothesis and indicates that CHS might affect the ATP level and glycerol-3-phosphate shuttles, which are a crossroad of glycolysis, fatty acid metabolism, and OXPHOS [95,96,97,98]. Furthermore, NADH can be reduced from NAD^+^ in the metabolic processes including glycolysis, β-oxidation, and the TCA cycle. Beyond its crucial role as a coenzyme in energy metabolism [99], NAD^+^ has been shown in recent years to play a vital role in stress resistance, DNA repair, cell death, and signal transduction [100,101,102,103]. Collectively, these data indicate that the increased levels of salicylate and the golden NAD^+^/NADH nucleotides may play a key role in broiler acclimatization and/or metabolic efficiency during CHS [104,105,106,107,108]. In addition to the abovementioned changes, CHS seemed to enhance endogenous duodenal UDP and CDP concentrations, indicating a modulation of nucleoside metabolism and duodenal intracellular uridine- and cytidine-homeostasis, which is in accordance with previous studies [109,110]. We interpret the increase in these nucleosides under our experimental conditions as an indication of metabolic shift and reprogramming to promote the use of nucleic acid precursors, likely via salvage pathway, to favor the pentose phosphate pathway (PPP) and counteract ROS and replenish ATP, which is energetically favored over expensive *de novo* synthesis. The involvement of the PPP was evidenced also by the increased abundance of riboflavin and D-erythrose 4-phosphate, which are intermediates in the PPP and precursors for the synthesis of the aromatic amino acids [111]. This, again, supports the notion of a metabolic steady-state adaptation under CHS, rerouting from glycolysis to the PPP as a metabolic transition to counteract heat stress [112,113,114]. Similarly, riboflavin, or vitamin B2, has been shown to play a crucial role in boosting the antioxidant and immune systems [115,116] under stress conditions. It is also possible that riboflavin could be involved in cellular oxidation and mitochondrial energy production via ETC [117] to sustain membrane stability and adequate energy-related cellular functions under CHS; however, further studies associated with the riboflavin/FAD cycle are warranted.

Partial least squares-discriminant analysis of the duodenal DA metabolites revealed a distinct separation between the HS and TN groups, indicating potential biomarkers and unique features predictive of HS. To gain further insights and identify biologically related molecule networks, we next mapped these DA metabolites to the IPA knowledge-base and analyzed the most enriched biological functions. As expected and in accordance with previous studies, both AHS and CHS affected purine nucleotide metabolism [118], urate biosynthesis/inosine 5′-phosphate degradation [119], and ascorbate recycling [120]. Interestingly, the adenosine nucleotide and dopamine degradation pathways were enriched in AHS; however, biosynthesis of UDP-D-xylose and UDP-D-glucuronate and pyrimidine salvage pathways were triggered in CHS. These changes are in consonance with previous studies [121,122,123,124,125]. Adenosine degradation could result in AMP or urea (via inosine) production, both of which were reported to be increased under acute heat stress conditions [126,127]. AMP increase is an indicator of ATP depletion, and urea increase has been reported as a metabolic strategy to compensate for reduced nutrient intake and increased energy expenditure under short-term heat stress exposure [126]. Dopamine has been reported to be produced by gastrointestinal tract by enteric neurons and intestinal epithelial cells in humans [128], and the dopaminergic system has been shown to be involved in stress response in many species, including avian species [122,129]. As described in previous paragraphs, IPA enrichment of the UDP-D-xylose and UDP-D-glucuronate biosynthesis and pyrimidine salvage pathways indicated a metabolic shift during CHS from glycolysis to PPP and sugar nucleotide oxidation pathways. In addition, although there is a paucity of literature in animals, UDP-D-glucuronate has been found to play a major role in drug metabolism and detoxification as well as neuroprotective function through dopamine and neurotransmitter glucuronidation [130]. In plants, however, UDP-xylose and UDP-D-glucuronate have been well established as key metabolites for stress tolerance [131,132,133].

The top-scoring diseases and disorders enriched by IPA from the DA metabolites were organismal and GI injury for both AHS and CHS; cancer and hematological, neurobiological, and psychological disorders in CHS; and ophthalmic, cardiovascular, developmental, and hereditary disorders in AHS. This is not surprising, as most of these disorders are associated with oxidative stress and mitochondrial dysfunction [134,135,136,137,138]. Of particular interest, heat stress has been reported to induce intestinal injury through the lysosome- and mitochondria-dependent pathway [139], and the identified DA metabolites in the present study could be a new vista opening for mechanistic and functional studies of the interaction of these metabolites and gut integrity under heat stress conditions. Similarly, the DA metabolites were mapped by IPA to several top molecular and cellular functions with protein synthesis, cell death and survival, and free radical scavenging enriched in CHS, while small molecule biochemistry, nucleic acid and carbohydrate metabolisms, DNA damage, and energy production were specific to AHS. This is also in agreement with previous mammalian studies [140,141,142,143,144,145,146] and suggests that the metabolic reprogramming and the duodenal metabolomic response pattern to environmental HS are duration-dependent. It seems that HS triggers different cellular mechanisms from favoring stress-related metabolites for averting DNA damage to adaptation/acclimatization-associated metabolites for survival.

The IPA regulatory networks identified several upstream regulators and downstream mediators. Although IPA predicted an activation of *CA9* in CHS (z score of 2) and *HDAC11* in PF (z score of 2), real-time quantitative PCR showed that these genes were downregulated by both heat stress conditions, indicating a potential feedback loop. Both *CA9* and *HDAC11* have been shown to be involved in mitochondrial function and many anomalies including stress, hypoxia and alkalosis [147,148,149,150], all of which are associated with heat stress [31,151,152]. In support of IPA-network prediction, molecular analyses demonstrated that CHS affected the duodenal expression of *MAPK1*, *MAPK14*, *MAPK9*, *SOD1*, and *SOD2* genes. The roles of these pathways in oxidative stress and cellular signaling are well established. SODs are key antioxidant enzymes that catalyze the dismutation of superoxide radicals to hydrogen peroxide [153]. Their reduced expression in our study is puzzling because most of the curated literature reported an increased activity under short-term heat stress exposure [154,155]. Although speculative, it is possible that the reduced SOD expression is associated with an exhaustion of the antioxidant defense system [156,157,158] in stabilizing duodenum integrity and adapting to CHS. Similarly, it has been shown that cells respond to high temperature by activating MAPKs [159,160,161], which are known as key mediators of stress responses. Their reduced expression in our experimental conditions suggests that the birds were adapted to CHS [162,163,164,165]. Furthermore, the CHS-induced metabolites have been shown to affect MAPKs. Indeed, salicylate, NAD^+^, NADH, and nucleosides were found to regulate the expression of MAPKs [166,167,168,169], which in turn have been reported to regulate metabolic reprogramming [170]. It is, however, worth mentioning that we measured only mRNA levels, and it is conceivable that the protein levels, enzymatic activity, and/or posttranslational modifications (phosphorylation) of these SODs were affected differently.

In conclusion, this study is the first to use a high-throughput mass spectrometric metabolomics approach to probe the heat stress-induced duodenal metabolic changes in broilers. Our results demonstrated that, independently of feed intake depression, HS induced significant changes in metabolic pathways in a duration-dependent manner and identified potential duodenal markers for HS. Although this data-driven approach was not designed to provide mechanistic and functional evidence, the IPA and molecular analyses provided additional understanding of HS responses in broiler duodenum and opened a new vista for future investigations.

## Figures and Tables

**Table 1:** Oligonucleotide real-time qPCR primers.

Gene	Accession Number ^a^	Primer Sequence (5′→3′)	Orientation	Product Size (bp)
*CA9* *HDAC11* *CPT1* *MAPK3* *MAPK1* *MAPK14* *MAPK9* *SOD1* *SOD2* *18S*	XM_004937157 NM_001277141 AY675193 NM_204150 AY033635 XM_419263 NM_205095 NM_205064 NM_204211 AF173612	GGGATGTGCTTGCTGTGCTAT AGGAAAGCCAGCATTGTGATG ACCAGTCCTCTTTCTTCCCAACT GGGTTCGCAGAGGTTTCAAA GCCCTGATGCCTTCATTCAA ATTTTCCCATGTCTCGGTAGTGA CGGACCATGATCACACAGGAT CAGGAGCCCTGTACCAACGT CGGACCATGATCACACAGGAT CAGGAGCCCTGTACCAACGT AGCTGGAGATTGAGGAATGGAA CGGTGGCACAAAGCTGATTA GCCGATGATCAGCCAGGAT GGCCCAATGGAAGCAAGAG TGGCTTCCATGTGCATGAAT AGCACCTGCGCTGGTACAC GCTGGAGCCCCACATCAGT GGTGGCGTGGTGTTTGCT TCCCCTCCCGTTACTTGGAT GCGCTCGTCGGCATGTA	Forward Reverse Forward Reverse Forward Reverse Forward Reverse Forward Reverse Forward Reverse Forward Reverse Forward Reverse Forward Reverse Forward Reverse	58 63 60 63 63 62 62 58 61 60

^a^ Accession numbers refer to Genbank (NCBI). *CA9*, carbonic anhydrase 9; *HDAC 11*, histone deacetylase 11; *MAPK*, mitogen-activated protein kinase; *SOD*, superoxide dismutase.

**Table 2:** Potential duodenal metabolic biomarkers ^1^.

	HMDB ID	PHS	PF	AHS	CHS
Cystathionine	HMDB0000099	**+**			**-**
N-Acetylornithine	HMDB0003357	**+**			**-**
Dihydroorotate	HMDB0003349	**+**		**-**	
Allantoate	HMDB0001209	**+**			
Trehalose	HMDB0000975	**+**			
Glucosamine	HMDB0001514	**+**	**+**		
sn-Glycerol 3-phosphate	HMDB0000126	**+**			**+**
NAD+	HMDB0000902	**+**			**+**
Cysteate	HMDB0002757	**-**	**-**		**+**
Inosine	HMDB0000195	**-**			
Acetylphosphate	HMDB0001494		**+**		**+**
Histidine	HMDB0000177		**+**	**-**	**-**
Homocysteic acid	HMDB0002205		**+**		
3-Phosphoserine	HMDB0000272		**+**		
N-Acetyl-beta-alanine	HMDB0061880		**+**		
Cystine	HMDB0000192		**+**		**-**
Methionine sulfoxide	HMDB0002005		**+**		
Arginine	HMDB0000517		**+**		
D-glucarate	HMDB0000663		**+**		
Xylose	HMDB0000098		**+**		
Glucose phosphate	HMDB0001254		**+**		**-**
Myo-inositol	HMDB0000211		**+**		
N-Acetylglucosamine 1/6-phosphate	HMDB0002817		**+**		
3-Phosphoglycerate	HMDB0000807		**+**		
2-Oxo-4-methylthiobutanoate	HMDB0001553		**+**		
FAD	HMDB0001248		**+**		**+**
Hypoxanthine	HMDB0000157		**+**		
Orotate	HMDB0000226		**+**	**-**	
Octulose bisphosphate	N/A		**+**		
Allantoin	HMDB0000462		**+**		
D-gluconate	HMDB0000625		**+**		**-**
Ribose phosphate	HMDB0001548		**+**		
Sedoheptulose 1/7-phosphate	HMDB0060509		**+**		
Aconitate	HMDB0000072		**+**		**-**
Pyridoxine	HMDB0000239		**+**		
AICAR	HMDB0001517		**-**	**-**	
Prephenate	HMDB0012283			**+**	**+**
pimelic acid	HMDB0000857			**+**	**+**
2-Isopropylmalate	HMDB0000402			**+**	**+**
3-Hydroxyisovalerate	HMDB0000754			**+**	
ADP-glucose	HMDB0006557			**+**	**+**
Homocarnosine	HMDB0000745			**-**	**-**
N-Carbamoyl-L-aspartate	HMDB0000828			**-**	
Homocysteine	HMDB0000742			**-**	
dAMP	HMDB0000905			**-**	
dTMP	HMDB0001227			**-**	
Glycinamide ribotide (GAR)	HMDB0002022			**-**	**-**
S-Adenosyl-L-homocysteine	HMDB0000939			**-**	
Homocitrulline	HMDB0000679			**-**	
Citrate/isocitrate	HMDB0000193			**-**	
Hydroxyphenylacetate	HMDB0000020				**+**
Salicylate	HMDB0000500				**+**
NADH	HMDB0001487				**+**
UDP	HMDB0000295				**+**
NADP+	HMDB0000217				**+**
CDP	HMDB0001546				**+**
D-Erythrose 4-phosphate	HMDB0001321				**+**
Riboflavin	HMDB0000244				**+**
N-Acetylputrescine	HMDB0002064				**-**
1-Methylhistidine	HMDB0000001				**-**
Phosphothreonine	HMDB0011185				**-**
Pantothenate	HMDB0000210				**-**

^1^ AHS, acute heat stress; CHS, chronic heat stress; HMDB, human metabolome database; PHS, preheat stress; PF, pair fed; +, increase; -, decrease.

**Table 3:** Top canonical pathways enriched by observed metabolite alterations in heat stress environment.

Canonical Pathways	Molecules	Treatments ^1^							
		PHS		AHS		CHS		PF	
		*p*-Value	Ratio	*p*-Value	Ratio	*p*-Value	Ratio	*p*-Value	Ratio
Purine Nucleotides Degradation	Hypoxanthine *, inosine, NAD^+^, NADH, uric acid, xanthosine, xanthosine monophosphate, adenosine ^†^, GMP ^†^	-	-	7.7 × 10^−8^	0.412	5.0 × 10^−7^	0.412	-	-
Urate Biosynthesis/Inosine 5′-phosphate degradation	NAD^+^, NADH, uric acid, xanthosine, xanthosine monophosphate	-	-	1.0 × 10^−6^	0.556	4.0 × 10^−6^	0.556	-	-
Adenosine Nucleotides Degradation	Hypoxanthine, inosine, NAD^+^, NADH, uric acid	-	-	3.8 × 10^−6^	0.455	-	-	-	-
Dopamine Degradation	3′,5′-ADP, 3,4-dihydroxyphenylacetic, NAD^+^, NADH	-	-	1.5 × 10^−5^	0.357	-	-	-	-
Ascorbate Recycling	Ascorbic acid, NAD+, NADH, NADP, glutathione ^†^	-	-	2.5 × 10^−5^	0.5	1.8 × 10^−6^	0.625	-	-
UDP-D-xylose and UDP-D-glucuronate Biosynthesis	NAD^+^, NADH, UDP-D-glucose, UDP-glucuronic acid	-	-	-	-	5.5 × 10^−6^	0.8	-	-
Salvage Pathways of Pyrimidine Deoxyribonucleotides	Deoxyuridine, dTMP, dUMP, thymidine, thymine, deoxycytidine ^‡^, uracil ^‡^	-	-	-	-	2.3 × 10^−5^	0.417	1.6 × 10^−6^	0.583
Glycine Betaine Degradation	Dimethylglycine, glycine, L-homocysteine, L-methionine, L-serine, Pyruvic acid, sarcosine	6.1 × 10^−7^	0.538	-	-	-	-	3.3 × 10^−6^	0.538
tRNA Charging	Glycine, L-arginine, l-asparagine, L-aspartic acid, L-histidine, L-methionine, L-phenylalanine, L-proline, l-serine, L-tryptophan, L-tyrosine	2.5 × 10^−6^	0.256	-	-	-	-	-	-
Creatine Biosynthesis	Creatine, glycine, glycocyamine, L-arginine, L-ornithine, S-adenosylhomocysteine ^‡^	4.3 × 10^−6^	0.714	-	-	-	-	3.0 × 10^−7^	0.857
Pyruvate Fermentation to Lactate	L-lactic acid, NAD^+^, NADH, Pyruvic acid	4.8 × 10^−6^	1	-	-	-	-	-	-

^1^ AHS, acute heat stress; CHS, chronic heat stress; HMDB, human metabolome database; PHS, preheat stress; PF, pair. * specific for AHS, † specific for CHS, ‡ specific for PF.

**Table 4:** Top diseases and disorders for DA metabolites in broiler duodenum under heat stress environment.

Diseases and Functions	Treatments ^1^							
	PHS		AHS		CHS		PF	
	*p*-Value	# Mol.	*p*-Value	# Mol.	*p*-Value	# Mol.	*p*-Value	# Mol.
Cancer	4.7 × 10^−2^–8.8 × 10^−9^	36	-	-	3.3 × 10^−2^–2.2 × 10^−5^	22	4.9 × 10^−2^–4.7 × 10^−10^	45
Organismal Injury and abnormalities	4.7 × 10^−2^–8.8 × 10^−9^	51	4.9 × 10^−2^–6.5 × 10^−4^	23	3.3 × 10^−2^–2.2 × 10^−5^	29	4.9 × 10^−2^–4.7 × 10^−10^	70
Hepatic system disease	4.7 × 10^−2^–1.7 × 10^−5^	22	-	-	-	-	-	-
Hematological disease	4.7 × 10^−2^–1.3 × 10^−4^	13	-		3.3 × 10^−2^–2.9 × 10^−4^	8	-	-
Ophthalmic disease	-	-	2.5 × 10^−2^–6.5 × 10^−4^	3	-	-	-	-
Cardiovascular disease	-	-	4.8 × 10^−2^–1.9 × 10^−3^	3	-	-	-	-
Developmental disorders	-	-	4.2 × 10^−2^–1.9 × 10^−3^	4	-	-	-	-
Hereditary disorders	-	-	4.2 × 10^−2^–1.9 × 10^−3^	6	-	-	-	-
Neurological disease	-	-	-	-	3.3 × 10^−2^–1.2 × 10^−4^	21	-	-
Psychological disorders	-	-	-	-	3.3 × 10^−2^–8.3 × 10^−4^	15	-	-
Inflammatory disease	-	-	-	-	-	-	4.9 × 10^−2^–1.4 × 10^−6^	21
Inflammatory response	-	-	-	-	-	-	4.7 × 10^−2^–1.4 × 10^−6^	32

^1^ AHS, acute heat stress; CHS, chronic heat stress; PHS, preheat stress; PF, pair fed.

**Table 5:** Top molecular and cellular functions for DA metabolites in broiler duodenum under heat stress environment.

	Treatments ^1^							
	PHS		AHS		CHS		PF	
Molecular and Cellular Functions	*p*-Value	# Mol.	*p*-Value	# Mol.	*p*-Value	# Mol.	*p*-Value	# Mol.
Amino acid metabolism	4.7 × 10^−2^–1.1 × 10^−8^	27	-	-	-	-	4.9 × 10^−2^–8.9 × 10^−9^	31
Molecular transport	4.7 × 10^−2^–1.1 × 10^−8^	37	-	-	-	-	4.9 × 10^−2^–2.5 × 10^−8^	48
Small molecule biochemistry	4.7 × 10^−2^–1.1 × 10^−8^	44	4.6 × 10^−2^–8.4 × 10^−5^	28	-	-	4.9 × 10^−2^–2.5 × 10^−8^	58
Protein synthesis	2.6 × 10^−2^–1.7 × 10^−7^	18	-	-	3.3 × 10^−2^–2.0 × 10^−4^	11	1.4 × 10^−2^–3.6 × 10^−7^	22
Cell death and survival	4.7 × 10^−2^–7.5 × 10^−7^	36	-		3.3 × 10^−2^–1.2 × 10^−4^	26	-	-
Nucleic acid metabolism	-	-	4.5 × 10^−2^–1.8 × 10^−5^	21	-	-	-	-
DNA replication, damage, and repair	-	-	4.2 × 10^−2^–3.0 × 10^−4^	11	-	-	-	-
Energy production	-	-	4.2 × 10^−2^–3.0 × 10^−4^	12	-	-	-	-
Carbohydrate metabolism	-	-	4.3 × 10^−2^–6.5 × 10^−4^	10	-	-	-	-
Free radical scavenging	-	-	-	-	3.3 × 10^−2^–1.4 × 10^−4^	12	-	-

^1^ AHS, acute heat stress; CHS, chronic heat stress; PHS, preheat stress; PF, pair fed.

**Table 6:** Top upstream regulators for DA metabolites in broiler duodenum under heat stress environment.

Upstream Regulators ^2^	Treatments ^1^							
	PHS		AHS		CHS		PF	
	*p*-Value	Z Score	*p*-Value	Z Score	*p*-Value	Z Score	*p*-Value	Z Score
GNMT	4.1 × 10^−4^	−2.23	-	-	-	-	8.0 × 10^−7^	−2.82
CPT1B	1.9 × 10^−9^	−2.49	1.3 × 10^−4^	−2.44	1.1 × 10^−5^	−2.82	1.9 × 10^−17^	−3.71
GATA4	2.8 × 10^−6^	2.82	-	-	-	-	1.8 × 10^−4^	2.64
MMP11	2.2 × 10^−5^	2.00	-	-	-	-	-	-
IL37	-	-	-		9.9 × 10^−4^	−2.23	2.9 × 10^−10^	−2.49
CA9	1.5 × 10^−11^	3.31	-	-	1.6 × 10^−3^	2.00	6.9 × 10^−9^	3.16
HDAC11	2.1 × 10^−4^	2.00	-	-	-	-	5.9 × 10^−4^	2.0
CTH			7.9 × 10^−5^	−2.0	-	-	-	-

^1^ AHS, acute heat stress; CHS, chronic heat stress; PHS, preheat stress; PF, pair fed; ^2^ CA9, Carbonic anhydrase; CPT1B, Carnitine palmitoyltransferase 1B; CTH, Cystathionine gamma-lyase; GATA4, GATA binding protein 4; GNMT, glycine N-methyltransferase; HDAC11, histone deacetylase 11; IL37, interleukin 37; MMP11, matrix metallopeptidase 11.

## Data Availability

Original datasets from all studied groups have been submitted to EMBL-EBI MetaboLights database, DOI: 10.1093/nar/gkz1019, PMID:31691833) with the identifier MTBLS4513 (https://www.ebi.ac.uk/metabolights/MTBLS4513).

## Supplementary Materials

The following supporting information can be downloaded at: https://www.mdpi.com/article/10.3390/ani12111337/s1, Table S1. Duodenal metabolite profile in heat stressed broilers.
