# Supplementary material for: Duodenal Metabolic Profile Changes in Heat-Stressed Broilers
Source: Animals (Basel). 2022 May 24;12(11):1337. doi: 10.3390/ani12111337 (PMC9179521; doi:10.3390/ani12111337)
Supplement: Supplementary file 1 [file animals-12-01337-s001.zip › animals-1679041-supplementary.pdf]

Table S1: Duodenal metabolite profile in heat stressed broilers.

Metabolites		Treatments[1]							
		PHS		PF		AHS		CHS	
Name	HMDB ID	Fold change	P value	Fold change	P value	Fold change	P value	Fold change	P value
Creatinine	HMDB0000064	0.96655536	0.915	1.24686587	0.486	0.95406314	0.903	1.1725837	0.665
homocarnosine	HMDB0000745	0.94028219	0.741	1.07129425	0.732	0.43087425	0.006	0.46603252	0.009
N-Acetylputrescine	HMDB0002064	1.344205	0.234	0.76597183	0.260	1.05721147	0.863	0.45135191	0.011
Prephenate	HMDB0012283	0.90100995	0.611	1.14744777	0.340	1.87210272	0.0008	1.66112409	0.023
Sulfolactate	HMDB0060176	0.81745362	0.310	1.09804041	0.368	1.0890293	0.649	1.3040037	0.334
Cystathionine	HMDB0000099	1.3608387	**0.067**	1.08793873	0.583	0.93242899	0.728	0.69619174	0.037
N-Carbamoyl-L-aspartate	HMDB0000828	1.08478594	0.776	1.38954548	0.282	0.53971903	0.029	0.79923533	0.264
Glucosamine phosphate	HMDB0001254	0.90605269	0.297	0.8685643	0.222	0.8374859	0.1599	0.92035224	0.636
Indole	HMDB0000738	1.17353637	0.643	1.32599931	0.319	0.87942085	0.661	1.07657229	0.792
Phenyllactic acid	HMDB0000779	0.36231429	0.100	0.82962103	0.659	1.55459106	0.221	1.7950216	0.276
Hydroxyphenylacetate	HMDB0000020	0.97989133	0.947	1.27719445	0.333	1.22574838	0.435	1.98249576	**0.064**
Phenylpyruvate	HMDB0000205	1.13683817	0.625	1.29032049	0.293	1.4044984	0.200	1.34858801	0.417
Salicylate	HMDB0000500	1.01575287	0.952	1.2731662	0.171	1.20462138	0.292	1.43919306	0.089
1-Methylhistidine	HMDB0000001	0.87600227	0.534	0.91001587	0.673	0.66849917	0.207	0.51778369	0.030
Acetylphosphate	HMDB0001494	2.08023411	0.170	1.95477928	0.017	1.5652178	0.381	1.71627514	0.062
2-Aminoadipate	HMDB0000510	1.00489719	0.981	1.30002818	0.202	0.91654777	0.674	1.18565023	0.477
Dimethylglycine	HMDB0000092	1.01080305	0.959	1.21758452	0.362	0.83703181	0.489	0.83521251	0.514
Glutamate	HMDB0060475	1.00561491	0.986	1.17085975	0.621	0.89757491	0.724	0.93835462	0.830
Glutamine	HMDB0003423	1.00071336	0.997	1.20697788	0.488	0.74880919	0.343	0.65872317	0.178
Glutathione	HMDB0000125	1.05409716	0.757	1.0827725	0.649	0.99244752	0.961	1.12189531	0.488
Glutathione disulfide	HMDB0003337	0.8248086	0.524	1.08239646	0.745	0.8782418	0.499	0.90743092	0.638
Guanidoacetic acid	HMDB0000128	1.34026335	0.480	1.64911235	0.345	1.74171342	0.314	3.55765398	0.221
Histidine	HMDB0000177	1.20156206	0.261	1.36946218	0.036	0.69343377	**0.077**	0.66554784	0.034
Homocysteic acid	HMDB0002205	1.08747363	0.642	1.59128226	0.034	1.14894172	0.507	1.33525964	0.142
Homoserine/Threonine	HMDB0000719	1.21445626	0.497	1.38578159	0.239	0.85185115	0.581	0.80509304	0.447
3-Phosphoserine	HMDB0000272	0.95060688	0.790	1.67847365	0.004	0.96311705	0.820	0.84367791	0.296

Methionine	HMDB0000696	1.13027432	0.552	1.27620057	0.218	0.79104675	0.305	0.77505945	0.233
N-Acetyl-beta-alanine	HMDB0061880	1.23863293	0.348	1.54287876	0.033	1.07316306	0.687	0.99651003	0.982
N-Acetylglutamine	HMDB0006029	1.03268364	0.857	1.24385558	0.128	0.96177787	0.805	0.95125294	0.821
N-Acetylornithine	HMDB0003357	1.30774726	0.023	0.9785113	0.866	0.93231255	0.709	0.7560973	0.042
Ophthalmate	HMDB0005765	0.8024419	0.367	1.14905635	0.538	0.92846819	0.735	0.76772075	0.280
Phosphothreonine	HMDB0011185	0.94068717	0.891	1.59075082	0.232	0.71587033	0.430	0.34186595	0.041
Pyroglutamic acid	HMDB0000267	1.10533802	0.630	1.29226131	0.133	1.15559484	0.442	0.98774653	0.950
Aspartate	HMDB0000191	1.02802534	0.911	0.95807107	0.862	1.02889983	0.907	1.12948069	0.618
Cystine	HMDB0000192	0.76789667	0.105	1.41855498	0.0003	0.99343771	0.964	0.74064432	0.026
Homocysteine	HMDB0000742	1.07269243	0.607	1.25108464	0.263	0.7182996	0.044	0.89928293	0.487
Hydroxyproline	HMDB0000725	1.13269841	0.562	1.1126074	0.670	0.78792726	0.345	0.90059653	0.662
Lysine	HMDB0003405	1.24558058	0.403	1.43671949	0.183	0.82546952	0.513	0.98471387	0.952
Methionine sulfoxide	HMDB0002005	1.19540046	0.405	1.47869181	0.029	0.72487692	0.171	0.69573133	0.141
Ornithine	HMDB0000214	1.23681734	0.326	1.48077095	0.102	0.82771794	0.468	0.93656065	0.774
Phenylalanine	HMDB0000159	1.05523259	0.803	1.21378416	0.304	0.99060378	0.955	1.05959776	0.712
Proline	HMDB0000162	1.11337282	0.642	1.3213653	0.181	0.76484182	0.271	0.75170315	0.231
Tyrosine	HMDB0000158	1.07784032	0.692	1.07976218	0.689	0.76851715	0.175	0.87696828	0.479
Valine/betaine	HMDB0240571	1.02632544	0.871	1.16945065	0.254	0.79855665	0.193	0.83362339	0.246
Acetyllysine	HMDB0000206	1.21738548	0.544	0.91406972	0.772	1.1170347	0.765	0.79908673	0.508
Alanine/Sarcosine	HMDB0000271	1.07284368	0.768	1.25038229	0.353	0.76228495	0.310	0.90566095	0.705
Asparagine	HMDB0000168	1.1904799	0.501	1.38091848	0.171	0.72109794	0.228	0.68189144	0.160
Citrulline	HMDB0000904	0.93606097	0.767	1.268649	0.316	0.86184763	0.544	0.91905127	0.726
Creatine	HMDB0000064	1.00381927	0.979	0.97380548	0.879	0.89648474	0.607	1.0977941	0.632
Cysteate	HMDB0002757	0.76929045	**0.068**	0.78513514	**0.051**	1.216111	0.2898	1.59705348	**0.062**
Dihydroorotate	HMDB0003349	1.4799577	**0.088**	1.35355887	0.101	0.55079754	0.038	1.04577955	0.867
Leucine/Isoleucine	HMDB0028932	1.06111515	0.755	1.15048807	0.414	0.82814108	0.339	0.85454517	0.386
N-Acetylglutamate	HMDB0001138	0.98312475	0.941	1.15216463	0.493	1.08939903	0.5971	1.09316396	0.602
Serine	HMDB0000187	1.17658849	0.548	1.50022121	0.145	0.838493	0.5384	0.82614129	0.507
Allantoate	HMDB0001209	1.31510817	**0.068**	1.18082488	0.197	1.05784086	0.665	1.045579727	0.653
Histamine	HMDB0000870	0.61178283	0.152	0.93075094	0.814	0.68958239	0.289	0.773458159	0.441

Tryptophan	HMDB0000929	1.09157286	0.665	1.27168574	0.155	0.97194484	0.855	1.02541611	0.868
aminoisobutyric acid	HMDB0001906	1.00955013	0.963	1.21122082	0.366	0.83106727	0.465	0.82559092	0.481
Arginine	HMDB0000517	1.32740096	0.101	1.48600013	0.046	0.80181445	0.294	0.88288066	0.460
Cysteine	HMDB0000574	0.90924602	0.374	1.25460834	0.154	0.92324273	0.632	0.838105581	0.255
Cholate	HMDB000019	0.34234223	0.236	0.43418777	0.299	1.73221356	0.496	0.800438666	0.730
Glycodeoxycholate	HMDB0000064	0.4578428	0.264	0.81737149	0.772	2.19859406	0.214	0.65075186	0.486
Taurodeoxycholate	HMDB0000896	0.77044571	0.437	0.99780429	0.994	1.01805674	0.947	0.932236913	0.802
Glycine	HMDB0000123	1.15389616	0.589	1.43603413	0.172	0.84065556	0.539	0.827054447	0.500
Taurine	HMDB0000251	1.08955669	0.599	1.08151979	0.676	0.9250556	0.723	1.081210867	0.731
5-Hydroxyindoleacetic acid (5-HIAA)	HMDB0000763	0.74216409	0.375	1.36667756	0.263	0.76866054	0.414	0.808480512	0.539
D-Glucarate	HMDB0000663	1.77029198	0.253	1.74762173	0.034	1.09917966	0.774	0.68193776	0.183
Trehalose/Sucrose	HMDB0000975	2.79732234	0.0456	1.41773619	0.315	0.63668	0.286	1.129217759	0.856
Xylitol	HMDB0002917	1.06154062	0.747	1.32109409	0.181	0.87515636	0.52	0.900287005	0.643
xylose	HMDB0000098	1.11565236	0.598	1.5028114	0.035	0.83362648	0.324	0.77934895	0.271
Fructose 1,6-bisphosphate	HMDB0001058	0.81238308	0.520	1.18782412	0.560	1.07570705	0.802	0.951902921	0.842
Glucosamine	HMDB0001514	1.53329968	**0.079**	1.61586491	**0.054**	0.68461431	0.187	0.734551983	0.280
Glucose phosphate	HMDB0001254	1.12043924	0.617	1.56598558	0.017	0.75673037	0.179	0.50176536	0.006
myo-Inositol	HMDB0000211	1.26328461	0.106	1.32462226	0.018	0.94132428	0.672	0.901442077	0.373
N-Acetylglucosamine	HMDB0000803	0.97463201	0.799	1.13894579	0.339	0.88192781	0.280	1.070479993	0.676
N-Acetylglucosamine 1/6-phosphate	HMDB0002817	0.97542441	0.810	1.34551344	0.021	0.89082181	0.397	0.98612521	0.915
Sedoheptoluse bisphosphate	HMDB0060274	1.8427042	0.451	2.00400794	0.325	1.54665108	0.529	1.530739048	0.526
sn-Glycerol 3-phosphate	HMDB0000126	1.72734159	0.024	0.96464182	0.849	0.88283593	0.679	1.335895751	**0.091**
Glycerone phosphate	HMDB0001473	1.07446762	0.708	1.22192296	0.233	0.88571524	0.483	0.78679935	0.195
Oxaloacetate	HMDB0000223	0.899186	0.591	1.33141428	0.101	0.99118846	0.934	1.184326607	0.185
Phosphoenolpyruvate	HMDB0000263	1.26178666	0.505	1.50079166	0.110	1.22549339	0.268	1.193714697	0.405
Pyruvate	HMDB0000243	1.09383166	0.677	1.21782881	0.428	0.89755519	0.603	0.902218894	0.583
Lactate	HMDB0000190	1.11412355	0.769	1.23849631	0.546	1.00213576	0.995	1.119907453	0.742
3-Phosphoglycerate	HMDB0000807	1.84152187	0.227	1.81107958	0.040	1.24938377	0.606	1.363340111	0.228
D-Glyceraldehdye 3-phosphate	HMDB0001112	1.09524098	0.730	1.32751715	0.198	0.91217132	0.653	0.839325596	0.456
2-Hydroxy-2-methylsuccinate	HMDB0000426	1.19221354	0.632	1.22595995	0.491	1.13301656	0.641	1.27211162	0.392

aminocaproic acid	HMDB0001901	1.06031708	0.755	1.14873849	0.414	0.82756447	0.333	0.852733082	0.375
Carnitine	HMDB0000062	1.02257605	0.915	1.10604984	0.563	0.77168252	0.254	0.7622147	0.204
Hydroxyisocaproic acid	HMDB0002064	0.63154008	0.174	0.9368038	0.804	1.38731283	0.212	1.575392723	0.357
methyl glutaric acid	HMDB0000752	0.72956019	0.209	1.21001862	0.480	1.148052	0.431	1.216893878	0.456
methyl succinic acid	HMDB0001844	1.24014235	0.635	1.18169139	0.780	0.5689329	0.142	1.115955771	0.853
pimelic acid	HMDB0000857	0.96728856	0.894	1.16606198	0.476	1.53772979	0.044	1.572743675	**0.078**
2-Isopropylmalate	HMDB0000402	1.13374009	0.443	1.01743561	0.888	1.44895644	0.027	1.622991594	**0.052**
Jasmonate	HMDB0032797	0.93011477	0.827	1.38050472	0.214	1.12180102	0.646	1.626904781	0.234
Cholesterol sulfate	HMDB0000653	0.08432358	0.351	1.05596851	0.962	0.28835899	0.466	0.31190322	0.491
2-Oxo-4-methylthiobutanoate	HMDB0001553	1.1542723	0.663	2.59898938	0.025	0.91316037	0.705	0.71167847	0.221
3-Hydroxyisovalerate	HMDB0000754	0.83278506	0.468	1.37879848	0.162	1.83011574	0.042	1.01579858	0.948
3,4-Dihydroxyphenylacetate (DOPAC)	HMDB0001336	0.96881363	0.905	1.2822162	0.304	1.18965555	0.440	1.53346269	0.242
Deoxyuridine	HMDB0000012	1.20915987	0.293	1.18802643	0.390	0.80475656	0.236	1.35432841	0.317
CDP-ethanolamine	HMDB0001564	1.06207281	0.733	0.90049369	0.598	0.91446461	0.634	0.99724868	0.990
dAMP	HMDB0000905	0.95869494	0.883	0.7701777	0.171	0.65949428	0.018	1.16030832	0.251
dCMP	HMDB0001202	1.04499336	0.883	1.10706465	0.662	0.74755871	0.132	0.94890086	0.757
deoxycytidine	HMDB0000014	1.15053643	0.590	1.20907649	0.333	0.85552117	0.544	0.65919493	0.103
Deoxyinosine	HMDB0000071	1.19012578	0.653	0.81806634	0.618	1.21392979	0.574	1.48667818	0.376
Dephospho-CoA	HMDB0001373	1.52338786	0.451	1.64332381	0.253	1.36279921	0.383	1.2694941	0.515
1-Methyladenosine	HMDB0003331	1.37273017	0.394	1.61139654	0.207	1.07965609	0.848	0.88578656	0.773
dTMP	HMDB0001227	1.17628272	0.567	1.07804013	0.641	0.71020358	0.014	1.04233811	0.755
dUMP	HMDB0001409	1.45760109	0.244	1.14983356	0.498	0.72376029	0.110	1.09927443	0.617
FAD	HMDB0001248	0.89574323	0.675	1.59904283	0.012	1.38018746	0.115	1.39201538	**0.096**
GDP	HMDB0001201	0.84827582	0.593	1.09834314	0.732	1.03985914	0.883	1.01999856	0.929
Glycinamide ribotide (GAR)	HMDB0002022	1.62317464	0.575	0.26731501	0.100	0.10142476	**0.052**	0.23066371	**0.088**
Guanosine	HMDB0000133	0.80183895	0.192	1.03438255	0.840	0.99436564	0.968	0.80021374	0.204
Adenosine	HMDB0000050	0.95915765	0.897	0.84030734	0.611	0.82867973	0.571	1.04966533	0.853
Inosine	HMDB0000195	0.75918255	0.0289	1.25446243	0.104	1.02275712	0.876	0.79313474	0.106
NADH	HMDB0001487	9.82055167	0.173	1.63567009	0.258	26.9818304	0.223	16.5912984	0.006
Thymidine	HMDB0000273	1.46344645	0.151	1.02167626	0.927	0.99078186	0.953	1.69949413	0.115

UDP	HMDB0000295	1.28207576	0.509	1.35100203	0.350	1.07539132	0.815	1.68874413	**0.081**
UDP-glucose	HMDB0000286	0.99669205	0.991	1.26157583	0.236	1.00817641	0.968	1.21383605	0.388
UDP-glucuronate	HMDB0000935	0.71277284	0.274	0.88967114	0.705	0.60421929	0.139	1.07973934	0.791
UMP	HMDB0000288	0.93412825	0.772	1.13186463	0.432	0.8282491	0.217	0.96634973	0.817
Uridine	HMDB0000296	1.0728606	0.561	1.05521057	0.673	0.83828047	0.221	1.09279923	0.612
Xanthosine	HMDB0000299	0.96879407	0.879	1.1339723	0.430	1.07966127	0.594	1.05167787	0.696
Xanthosine 5'-phosphate	HMDB0001554	0.70068523	0.300	0.90790823	0.741	1.61769868	0.162	1.89267567	0.153
ADP-glucose	HMDB0006557	1.05061309	0.897	1.03076887	0.887	2.0109164	0.029	2.16720534	0.047
cAMP	HMDB0000058	0.96600755	0.872	0.93834675	0.728	1.06325812	0.657	1.14545645	0.258
FMN	HMDB0001520	1.86554144	0.533	1.82151743	0.291	6.20607402	0.176	1.0633604	0.919
ADP	HMDB0000061	0.93352877	0.838	1.13297347	0.647	1.05144094	0.850	1.16707367	0.501
AICAR	HMDB0001517	1.07355519	0.867	0.60949907	**0.052**	0.40646283	0.005	0.77965719	0.341
CMP	HMDB0000095	0.72333032	0.132	0.89720484	0.484	0.77508559	0.141	0.75820109	0.117
Deoxyadenosine	HMDB0000101	1.34628868	0.645	1.32345253	0.617	0.96402824	0.945	0.4602868	0.214
GMP	HMDB0001397	0.98809213	0.966	1.04467371	0.833	0.98046704	0.922	1.11307029	0.608
IMP	HMDB0015536	0.94124557	0.824	0.98717063	0.950	0.80550067	0.261	0.890779	0.604
NAD+	HMDB0000902	2.40736348	0.049	0.91798801	0.788	1.5612597	0.256	2.68573331	0.006
NADP+	HMDB0000217	1.44701414	0.478	1.2143402	0.437	1.31090534	0.246	1.82331148	0.036
S-Adenosyl-L-homocysteine	HMDB0000939	0.91755328	0.780	1.23352875	0.324	0.46825483	0.011	0.65452067	0.246
UDP-N-acetylglucosamine	HMDB0000290	0.99207261	0.976	1.22305528	0.281	0.98393331	0.925	1.21746876	0.326
AMP/dGMP	HMDB0060465	0.94680531	0.856	1.02569248	0.915	0.93285954	0.762	1.1698026	0.509
Aminoimidazole ribotide (AIR)	HMDB0001235	0.98142909	0.975	0.57129707	0.411	0.14280231	0.102	0.39595273	0.240
7-Methylguanosine	HMDB0001107	0.96311991	0.921	0.69515594	0.311	1.56819548	0.296	0.841436	0.612
Guanine	HMDB0000132	0.87174255	0.602	0.7539873	0.229	0.92639631	0.738	0.85022647	0.591
Hypoxanthine	HMDB0000157	0.84492842	0.323	1.58201516	**0.066**	1.08031575	0.742	0.84717837	0.385
Orotate	HMDB0000226	0.91737804	0.646	1.56139665	0.009	0.51264246	0.001	3.67217132	0.270
Thymine	HMDB0000262	0.99574108	0.991	1.40939628	0.360	1.07136736	0.863	1.8662221	0.390
CDP	HMDB0001546	0.98385135	0.972	1.14591354	0.742	1.16597443	0.756	1.81773666	**0.090**
phosphorylethanolamine	HMDB0000224	1.1333028	0.547	1.00203163	0.992	0.907289	0.648	1.11532428	0.679
Octulose bisphosphate	N/A	0.77465941	0.472	3.15834038	0.008	2.18963423	0.186	0.584397522	0.135

Pikatropin	N/A	1.3739234	0.477	1.66077891	0.138	1.37849316	0.353	2.176076587	0.170
2-hydroxyglutaric acid	HMDB0000606	1.19543197	0.630	1.22714533	0.494	1.13481533	0.640	1.277553553	0.388
Glycolate	HMDB0000115	1.07379813	0.873	1.22275895	0.625	1.12255383	0.779	1.316026441	0.507
homocitrulline	HMDB0000679	1.01885255	0.926	1.18610005	0.512	0.64464617	**0.060**	0.790473543	0.238
glutaric acid	HMDB0000661	1.29942261	0.568	1.24003926	0.724	0.58094493	0.138	1.162472898	0.803
Nicotinamide	HMDB0001406	0.81694816	0.243	1.04144046	0.828	0.90041228	0.548	0.843006939	0.294
Allantoin	HMDB0000462	1.17760726	0.394	1.48555088	**0.050**	0.89834371	0.628	0.817640192	0.305
Uric acid	HMDB0000289	1.14227237	0.564	1.19790833	0.401	1.12115282	0.558	1.54813179	0.126
Uracil	HMDB0000300	0.90360188	0.438	1.26485511	0.171	0.84345433	0.357	0.77717537	0.132
Homovanillic acid (HVA)	HMDB0000118	0.40059831	0.116	0.83160678	0.665	1.44702127	0.344	1.38322992	0.459
2-Dehydro-D-gluconate	HMDB0011732	0.98185129	0.900	1.18183091	0.151	0.9063937	0.306	1.04546241	0.749
Glycerate	HMDB0000139	0.91185431	0.721	1.45776407	0.118	1.1737291	0.557	0.93110074	0.771
6-Phospho-D-gluconate	HMDB0001316	1.19473732	0.568	1.14255536	0.564	0.72025778	0.140	0.7702669	0.222
D-Erythrose 4-phosphate	HMDB0001321	1.26914882	0.496	1.26113579	0.339	1.47149792	0.228	2.470193478	0.015
D-Gluconate	HMDB0000625	1.22031214	0.175	1.35028373	0.015	0.91017314	0.543	0.793345451	0.038
Ribose phosphate	HMDB0001548	1.02649665	0.876	1.71472302	0.003	1.1626538	0.539	0.816229158	0.186
Sedoheptulose 1/7-phosphate	HMDB0060509	1.13820668	0.529	1.79460879	0.001	1.30555585	0.335	0.754331577	0.168
alpha-Ketoglutarate	HMDB0061388	1.91217322	0.355	1.41533137	0.184	0.76077648	0.346	0.883975568	0.589
Citrate/isocitrate	HMDB0000193	1.22713138	0.472	1.22683789	0.271	0.6699654	**0.074**	0.829853563	0.500
Fumarate	HMDB0000134	0.92759901	0.797	1.17675544	0.494	0.81107575	0.348	0.848636639	0.471
Malate	HMDB0000744	0.92809121	0.792	1.2257947	0.364	0.83759276	0.409	0.869622437	0.512
Succinate/Methylmalonate	HMDB0000202	1.30759651	0.438	0.85221733	0.483	0.88429866	0.585	1.43584621	0.135
Aconitate	HMDB0000072	1.3387742	0.284	1.52723214	0.006	0.73308673	0.159	0.63352078	0.044
Folate	HMDB0000121	0.57531058	0.361	1.05671381	0.912	0.80372608	0.681	0.59323101	0.411
Riboflavin	HMDB0000244	1.06878033	0.870	1.13539629	0.658	1.4820999	0.140	1.66532649	**0.088**
Biotin	HMDB0000030	1.04348997	0.909	1.74306427	0.270	1.16456589	0.649	1.44221967	0.268
Pantothenate	HMDB0000210	0.85705935	0.452	1.05110832	0.754	0.82014538	0.116	0.78807498	0.027
4-Pyridoxate	HMDB0000017	0.90544122	0.714	1.2096082	0.207	1.13040072	0.511	1.63051015	0.460
Ascorbate	HMDB0000044	1.10845151	0.126	0.88617294	0.254	1.04727385	0.521	1.10788159	0.123
Nicotinate	HMDB0001488	1.18945544	0.639	1.47258949	0.230	1.36245552	0.293	0.71740995	0.320

Pyridoxine	HMDB0000239	1.47042984	0.147	1.81680757	0.037	0.79671635	0.420	0.608844074	0.128

[1]AHS, acute heat stress; CHS, chronic heat stress; PHS, preheat stress; PF, pair fed
